# Dietary Flavonoids, Copper Intake, and Risk of Metabolic Syndrome in Chinese Adults

**DOI:** 10.3390/nu10080991

**Published:** 2018-07-29

**Authors:** Rongge Qu, Yubing Jia, Junyi Liu, Shanshan Jin, Tianshu Han, Lixin Na

**Affiliations:** National Key Discipline, Department of Nutrition and Food Hygiene, School of Public Health, Harbin Medical University, 157 Baojian Road, Harbin 150081, China; qurongge1992@163.com (R.Q.); 13045329318@163.com (Y.J.); Liujunyixq@163.com (J.L.); jinshanshan1723@sina.com (S.J.); snowcalendar@126.com (T.H.)

**Keywords:** flavonoids, copper, metabolic syndrome

## Abstract

The effects of flavonoids and copper (Cu) on metabolic syndrome (MetS) have been investigated separately, but no information exists about the joint associations between flavonoids and Cu on the risk of MetS in population studies. In this cross-sectional study, a total of 9108 people aged 20–75 years from the Harbin Cohort Study on Diet, Nutrition, and Chronic Non-Communicable Diseases (HDNNCDS) were included. Flavonoid intakes were calculated based on the flavonoid database created in our laboratory. Cu and other nutrient intakes were estimated using the Chinese Food Composition Table. Among all study subjects, a total of 2635 subjects (28.9%) met the diagnostic criteria for inclusion in the MetS group. Total flavonoids (fourth vs. first quartile, odds ratio (OR): 0.77, 95% confidence interval (CI) 0.66–0.90, P_trend_ = 0.002) and Cu (OR 0.81, 90% CI: 0.70–0.94, P_trend_ = 0.020) were inversely associated with the risk of MetS after adjusting for potential confounders. Higher flavonoid intake was more strongly associated with a lower risk of MetS with high levels of Cu intake (P_interaction_ = 0.008). Dose–response effects showed an L-shaped curve between the total intake of five flavonoids and the risk of MetS. These results suggest that higher flavonoid intake is associated with a lower risk of MetS, especially under high levels of Cu intake.

## 1. Introduction

Metabolic syndrome (MetS) is characterized by a cluster of metabolic abnormalities, including abdominal obesity, hyperglycemia, hypertension, and dyslipidemia [1]. MetS is a complicated interaction between genetic, metabolic, and environmental factors in which diet is a potent and modifiable environmental factor. Some diets and nutrients have been shown to play a protective role against the development of MetS [2,3]. As people’s interest in dietary life and health has increased, some diet and nutrients have been shown to play protective roles against the development of MetS, but the roles of flavonoids and copper in the MetS have not yet been clearly determined [4,5].

Flavonoids are a class of plant secondary metabolites. They are the most common group of polyphenolics in the human diet. Flavonoids are relatively abundant in fruits, vegetables, grains, herbs, and beverages, having a wide range of biochemical and pharmacological effects, such as anti-inflammatory and anti-proliferative actions [6,7]. Flavonoids are strong antioxidants and metal chelators with beneficial therapeutic characteristics, encouraging their development as candidates for targeting metal-induced diseases [8]. Copper (Cu) is an essential trace metal that is required for the catalysis of several important cellular enzymes. Some studies have shown that dietary Cu intake is significantly and inversely associated with MetS [9,10,11]. The flavonoid–copper (Cu) complex was reported to have anti-tumor properties by promoting the cleavage of plasmid DNA and inducing oxidative DNA damage [12,13,14]. So, we are interested in whether different levels of Cu intake can influence the relationship between flavonoids and MetS.

Therefore, the aim of this study was to clarify the association of dietary flavonoids and Cu with MetS and explore the interaction between dietary flavonoids and Cu in their effect on MetS in a large cross-sectional study of adult residents in urban Harbin, North China. We hypothesis that total flavonoids and Cu are all inversely associated with the risk of MetS, and their combination can significantly reduce this risk.

## 2. Materials and Methods

### 2.1. Study Population

**Figure 1:**
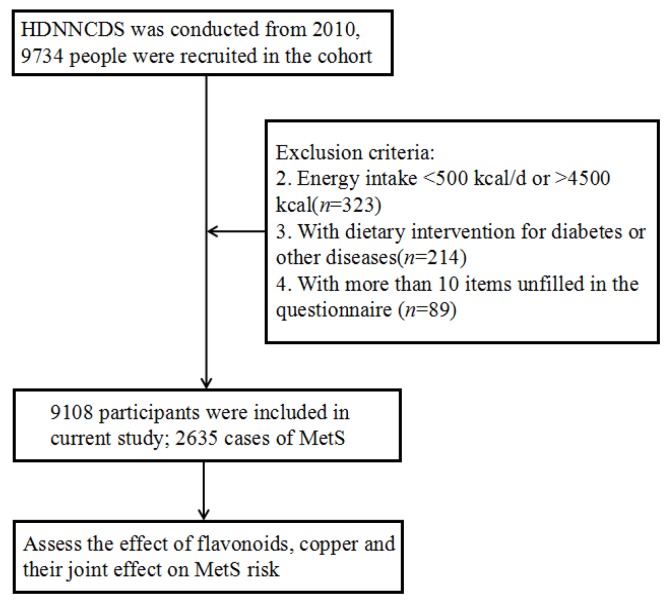
The flow diagram of this study. HDNNCDS is the Harbin Cohort Study on Diet, Nutrition, and Chronic Non-Communicable Diseases and MetS is metabolic syndrome.

Our study subjects were from the Harbin Cohort Study on Diet, Nutrition, and Chronic Non-Communicable Diseases (HDNNCDS) (Trial Registration: ChiCTR-ECH-12002721 at http://www. chictr.org.cn/showproj.aspx?proj=6833) launched in 2010 [15]. The HDNNCDS covered 7 urban administrative regions of Harbin. Each region was divided into 3 strata according to financial situation, and a total of 42 communities were randomly selected from each stratum in each administrative region by performing a stratified multi-stage random cluster sampling design. Residents who had lived in their communities for more than two years and did not have cancer or type I diabetes were included in our survey. A total of 9734 persons aged 20–75 years participated in the HDNNCDS. We excluded subjects at baseline who reported extreme values for total energy intake (<500 kcal/day or >4500 kcal/day) (*n* = 323) and who had undergone dietary intervention for diabetes or other diseases (*n* = 214) or who had more than 10 items unfilled in the questionnaire (*n* = 89). Finally, a total of 9108 subjects were eligible for analysis. The study protocol of HDNNCDS was approved by the Ethics Committee of Harbin Medical University, and written informed consent was provided by all subjects. The methods in this study were in accordance with the approved guidelines (Figure 1).

### 2.2. Questionnaire Data Collection

Detailed in-person interviews were administered by trained personnel using a structured questionnaire to collect information on demographic characteristics, dietary intake, lifestyle, and physical condition. The section on dietary intake was evaluated using the validated food frequency questionnaire (FFQ). A total of 103 food items were included in the questionnaire, which covered most of the commonly consumed food in urban Harbin. For each food item, the subjects were asked how frequently they had consumed that food over the preceding year, followed by a question on the amount consumed in liang (a unit of weight equal to 50 g) or mL (for liquid food items) per unit of time. Then, we used the amount of each item multiplied by the consumption frequency to obtain the daily intake of each food item. Cu and other nutrient intakes for each food item consumed were calculated by multiplying the nutrient content listed in the Chinese Food Composition Table [16]. The section on lifestyle and physical condition mainly included information about labor intensity, smoking, alcohol consumption, and taking medicines and health products over the past 12 months.

### 2.3. Dietary Flavonoid Assessment

The reverse phase high performance liquid chromatography (RP-HPLC) method was used to determine flavonoid levels among the food items commonly consumed in Harbin, China. A total of 41 food items in the questionnaire, including 3 potatoes and their products, 7 legumes and their products, 19 fresh vegetables, and 14 fresh fruits were assessed for flavonoid content. In the present study, we only quantified the content of the following flavonoids in plant species: three major flavonols—quercetin, kaempferol and isorhamnetin—and two major flavones—luteolin and apigenin—which have been most widely investigated in anti-carcinogenesis studies; the effects of other flavonoid subclasses on MetS were not considered [17]. Then, the detected flavonoid contents of each food item (flavonid-rich food) commonly consumed in Harbin city, multiplied by the reported consumption amount, as assessed by the FFQ, was considered to be the dietary flavonoid intake.

### 2.4. Anthropometric Measurement and Biochemical Assessment

Anthropometric measurements, including height, weight, and waist circumference, were taken by well-trained examiners, with subjects wearing light, thin clothing and no shoes. Body weight and height were measured to the nearest 0.1 kg and 0.1 cm, respectively. Body mass index (BMI) was calculated as weight (kg) divided by the square of the height in meters (m^2^). Systolic blood pressure (SBP) and diastolic blood pressure (DBP) were measured 3 times with a standard mercury sphygmomanometer on the right arm of each subject after a 10-min rest in a sitting position, and the mean values were used for analysis. Fasting and postprandial (2 h after drinking a 75-g glucose-containing water) blood samples were taken from all participants at baseline. Fasting plasma glucose (FPG), 2-h postprandial plasma glucose (PPG), blood lipids, including total cholesterol (TC), triglyceride (TG), low density lipoprotein cholesterol (LDL), and high density lipoprotein cholesterol (HDL), were measured using an automatic biochemistry analyzer (Hitachi, Tokyo, Japan).

### 2.5. Study Outcome Definition

Metabolic syndrome (MetS) is diagnosed according to the International Diabetes Federation (IDF) 2005 guidelines [18] as the presence of central obesity (waist circumference ≥90 cm for men and ≥80 cm for women) plus any two of the following conditions: (1) hypertriglyceridemia (TG ≥ 1.7 mmmol/L or specific treatment for this lipid abnormality); (2) low HDL cholesterol (HDL < 1.03 mmol/L in men or <1.29 mmol/L in women, or specific treatment for this lipid abnormality); (3) high blood pressure (SBP ≥ 130 mmHg or DBP ≥ 85 mmHg, or treatment of previously diagnosed hypertension); and (4) hyperglycemia (FPG ≥ 5.6 mmol/L or previously diagnosed type 2 diabetes).

### 2.6. Statistical Analysis

Data were presented as percentages for categorical variables and means ± standard deviations for continuous variables. Differences in sociodemographics, body composition, clinical and metabolic parameters, and dietary nutrient and flavonoid intakes between MetS subjects and control subjects were evaluated using *t*-tests for continuous variables and the Chi-square tests for categorical variables. A multivariate logistic regression analysis was used to estimate the adjusted odds ratios (OR) and 95% confidence intervals (CI) of the flavonoid and copper intake in predicting MetS. Flavonoid and Cu intakes in the model were standardized by energy (the crude dietary flavonoid or nutrient intake per 1000 kcal of total energy) and then divided into quartile categories based on the cumulative average. To determine the confounding factors in this study, MetS prevalence difference according to basic factors, such as age (years), sex (%), smoking (%), drinking (%), physical activity (%), and energy intake (kcal/day), was verified. For energy adjusted fat (g/kcal), fiber (g/kcal), protein (g/kcal), and carbohydrate (g/kcal), no significant differences in the MetS group were observed (energy adjusted fat, *p* = 0.394; energy adjusted fiber, *p* = 0.975; energy adjusted protein, *p* = 0.673; and energy adjusted carbohydrate, *p* = 0.607). Therefore, in our study, potential confounders included age, body mass index, sex, drinking, smoking, and physical activity intensity. Cubic splines were performed to evaluate the shape of the flavonoid–MetS relationship and to assess the dose–response relation. All statistical analyses were performed using SPSS v21.0 (Beijing Stats Data Co., Ltd., Beijing, China) and R 2.15.1 (http://www.r-project.org/). A two-sided *p* < 0.05 was considered statistically significant.

## 3. Results

### 3.1. General Characteristics

The sociodemographics, body composition, and clinical and metabolic parameters in both groups with and without MetS are shown in Table 1. Among this study’s population (*n* = 9108), 2635 (28.9%) individuals were MetS subjects meeting the International Diabetes Federation (IDF) 2005 guidelines. Compared with the control group, the MetS group were older, had a larger BMI and waist circumference, and worse blood glucose and lipid profiles (*p* < 0.01). A larger percentage of smokers was found in the MetS group (*p* < 0.01). Moreover, subjects with the highest flavonoid intake were more likely to engage in light physical activity (*p* < 0.05).

### 3.2. Dietary Nutrient and Flavonoid Intakes

The dietary nutrient and flavonoid intakes of the subjects from the groups both with and without MetS are shown in Table 2. The average daily intake of energy in the control group was significantly higher than in the MetS group (*p* < 0.05), but no differences were observed for protein, carbohydrate, fat, or fiber between the two groups. In addition, the dietary intakes of total flavonoid (mg/kcal) and Cu (ug/kcal) in the control group were significantly higher than those in the MetS group (*p* < 0.05). In terms of the subclasses of flavonoids, quercetin and luteolin intakes (mg/kcal) were also significantly higher than in the MetS group (*p* < 0.05).

### 3.3. Relationship between Flavonoid and Cu Intake, and MetS Risk

The relationships between flavonoid and Cu intake and the risk of MetS are shown in Table 3. Total flavonoid and Cu intake were strongly inversely associated with the risk of MetS after adjusting for age and sex (Mode 1: fourth vs. first quartile, OR = 0.72, 95% CI = 0.63–0.82, P_trend_ = 0.000 in flavonoid; OR = 0.79, 95% CI = 0.69–0.90, P_trend_ = 0.001 in Cu). The strength of this relationship decreased but remained significant after adjusting for Model 1 plus BMI, physical activity, drinking, and smoking (Model 2: OR = 0.77, 95% CI = 0.66–0.90, P_trend_ = 0.002 in flavonoid; OR = 0.81, 95% CI = 0.70–0.94, P_trend_ = 0.020 in Cu).

Flavonoid and Cu intakes in the model were first standardized by energy (the crude dietary flavonoids or nutrients intake per 1000 kcal total energy) and then divided into quartile categories based on the cumulative average. Data are presented as odds ratios (OR) and 95% CI of each quartile of flavonoid and copper intake. Model 1 included adjustment for sex and age, and Model 2 included Model 1 plus adjustment for BMI, drinking, smoking, and physical activity.

### 3.4. Joint Analyses of Flavonoid and Cu Intake on MetS Risk

In Table 4, flavonoid intake is divided into tertile levels according to the average energy-adjusted flavonoid intake. Compared with the lowest flavonoid intake group, the OR (95% CI) of MetS for the highest flavonoid intake group was 0.80 (0.70–0.91, P_trend_ = 0.003) after adjusting for potential confounders. Cu intake was divided into secondary levels according to the average energy-adjusted Cu intake. Compared with the lower Cu group, the OR (95% CI) of MetS for the higher Cu group was 0.85 (0.77–0.95, *p* = 0.003) after adjusting for potential confounders. Joint analyses showed that interactions occurred between total flavonoid and Cu intakes on MetS. The reverse association of total flavonoid intake with MetS risk was remarkably stronger under high levels of Cu intake (P_interaction_ = 0.000).

**Figure 2:**
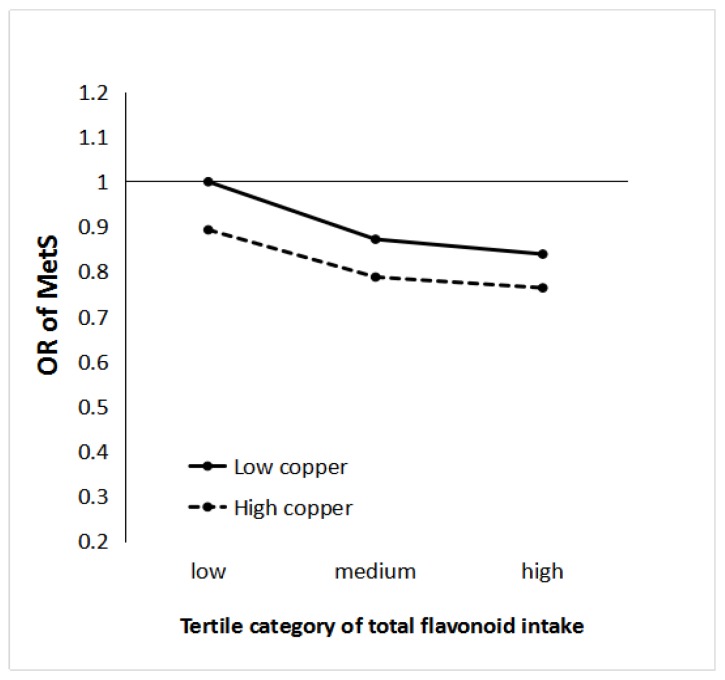
Joint associations of the odds ratio (OR) of flavonoid and copper on the risk of metabolic syndrome (MetS) (P_interaction_ = 0.000). Data represent the OR and 95% confidence intervals of different levels of flavonoid–copper intake adjusted for age, body mass index, sex, drinking, smoking, and physical activity. Tertile-specific point estimates are provided for low, medium, and high flavonoid intakes in tertile categories of low (solid line) and high (dashed line) copper intake.

In Figure 2, the OR (95% CI) of MetS under the high copper and high flavonoid patterns was 0.76 (0.66–0.89), compared with that under the low copper and low flavonoid pattern [1], representing a difference in relative risk of 24%.

### 3.5. Suggestions for Flavonoid Intake for the Prevention of MetS

**Figure 3:**
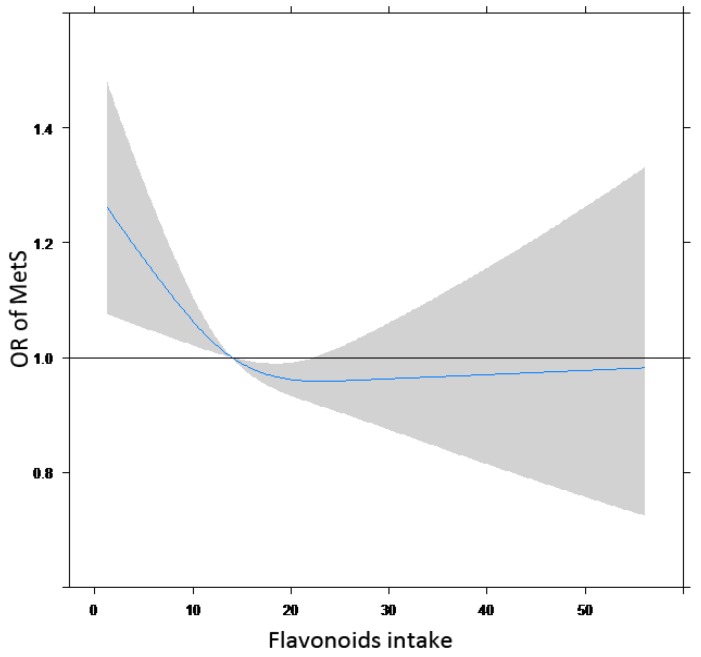
The approximated non-linear trend between the total intake of five flavonoids and the risk of MetS using restricted cubic spline. Data are presented as odds ratios (OR) and 95% confidence intervals of flavonoid intake adjusted for age, body mass index, sex, drinking, smoking, and physical activity.

In a dose–response analysis using cubic spline (Figure 3), we used the median total intake of flavonoids (14 mg/kcal) as the reference point, with three knots (5th, 50th, and 95th percentile) to approximate the relationship between the total intake of flavonoids and the risk of MetS. The relationship between flavonoid intake (continuously measured) and MetS risk was non-linear. We observed that a higher intake of flavonoids was associated with a decreased risk of MetS. The risk of MetS declined steadily as the total intake of the five flavonoids increased, until the flavonoid intake reached 23 g/kcal. The OR (95% CI) of MetS was 0.92 (0.76–1.00). At this point, the risk of MetS development was the lowest; however, the downtrend then increased slightly in an L-shape.

## 4. Discussion

In this study, we explored the association of dietary flavonoid and Cu intakes with the risk of MetS in a large urban cross-sectional study of Chinese adults living in Harbin, North China. We observed that higher intakes of flavonoids and Cu were significantly associated with a lower risk of MetS, with 23% and 19% reductions in the highest versus the lowest (reference) intake category, respectively. The reverse association of total flavonoids with MetS became much stronger in the context of a high copper intake. In addition, dose–response effects showed an L-shaped curve between the total intake of flavonoids and the risk of MetS.

Dietary intakes of total flavonoids have been reported to be inversely associated with metabolic syndrome among Polish adults [19]. In addition, a study found that higher consumption of total flavonoids was associated with a lower risk of MetS in Iranian adults [20]. In our study, we also found that total flavonoid intake was strongly inversely associated with the risk of MetS, even after adjusting for potential confounders, which was consistent with previous studies. The mechanisms for the beneficial effects of flavonoids against MetS may be their antioxidant and anti-inflammatory properties as well as their direct effects on endothelial function and nitric oxide (NO) bioavailability in the arterial vasculature [21,22].

Cu deficiency is not common, but Cu deficiency has been reported to increase HDL cholesterol levels in rats [23], blood cholesterol levels in adults [24], and even lead to arterial diseases, pigmentation loss, myocardial disease, and neurological effects [10]. Various studies have been completed on the relationship between Cu intake and MetS. Previous studies reported that Cu intake is related to MetS in states of insufficient or low Cu [25], whereas others reported that the association between Cu and MetS did not remain significant after adjustments [10]. Our results suggest that Cu is strongly inversely associated with the risk of MetS, even after adjusting for potential confounders. The mechanism for this relationship may be interpreted by Cu combining with superoxide dismutase (SOD), inhibiting the oxidation of cells, reducing free radicals, or through a reduction in glucose levels [10,11].

A variety of nutrients or bioactive substances in food may interact with each other during digestion, absorption, and metabolism in the body. An in vitro study reported that copper and flavonoids can form a metal ion complex. These metal chelating properties of flavonoids may play a role in metal-overload diseases and in all oxidative stress conditions involving a transition metal ion due to their anti-tumor properties [11,12,26,27]. However, we do not know whether these chelates of Cu and flavonoids can influence the effect of flavonoids on MetS in the human body. In our study, we observed that the flavonoid–MetS relationship was modestly modified by copper intake. Conversely, flavonoids’ inverse associations with MetS appeared stronger in the context of a high copper intake, suggesting that flavonoids combined with a variety of resources of other nutrients, such as copper, contribute to the lower risk we observed. Further, these data point to flavonoid intake as being only one aspect of a healthy diet, and to the benefits of a varied diet in obtaining adequate intake for ubiquitous nutrients such as copper. These points become more important in the context of popular dietary trends that recommend a balanced diet.

The mean intake of daily flavonoids for the total population was 34.68 mg/day, whereas the average intake of flavonoids in the US and Spain are 189.7 mg/day [28] and 313.26 mg/day [29], respectively, which are higher than our results. The highly variable average intake of flavonoids among studies in other countries and ours is partly because different flavonoid subclasses and different food resources were studied. According to our analysis, the inverse relationship between flavonoid intake and MetS (L-shaped) indicated that moving from a low to high intake of total flavonoids should be responsible for a reduction in the risk of MetS. However, the intensity of the reduction weakened and even slightly increased if more flavonoids were ingested. So, we recommend that eating flavonoid-rich foods in a reasonable range may help reduce the risk of MetS.

The strength of our study is that it is the first large sample, population-based, cross-sectional study that has analysed the effect of flavonoids, Cu, and their joint effects on MetS. We further assessed dose–response effects of flavonoid intake on MetS, providing more practical suggestions. In addition, the calculation of flavonoid intake was based on measured values of the food items consumed in Harbin which greatly improved the flavonoid intake estimation accuracy. Our study also had limitations. First, we only included 41 food items when calculating the amount of flavonoids. Our results for the mean dietary intake of flavonoids may be underestimated by the lack of food resources for tea, red wine, cocoa, etc., which are also main food sources of flavonoids. Second, only five flavonoids were included in the analysis based on the detected data in the earlier stage. The effects of other flavonoid subclasses on MetS were not considered.

## 5. Conclusions

In conclusion, individuals with higher intakes of flavonoids showed a lower MetS risk, with modestly stronger inverse associations observed in the presence of high copper intake. We suggest that eating flavonoid-rich foods combined with mineral intake may help to reduce the risk of metabolic syndrome. Further investigations including clinical trials and cohort studies are required to confirm these findings.

## Figures and Tables

**Table 1:** Selected characteristics of subjects from the with and without metabolic syndrome groups including sociodemographics, body composition, and clinical and metabolic parameters.

Variable	Control (*n* = 6473)	MetS (*n* = 2635)	*p*-Value
**Sociodemographics**			
Age (years)	49.33 ± 10.34	54.17 ± 9.34	0.000
Male, no. (%)	2266 (35.0)	978 (37.1)	0.057
Drinking, no. (%)	2140 (34.1)	889 (34.5)	0.747
Smoking, no. (%)			0.000
Yes	1064 (16.6)	500 (19.1)	
No	5174 (80.7)	1988 (76.0)	
Quit	175 (2.7)	127 (4.9)	
Physical activity, no. (%)			0.047
Light	5250 (82.9)	2189 (85.0)	
Middle	973 (15.4)	352 (13.7)	
Heavy	110 (1.7)	35 (1.4)	
**Body Composition and Clinical Parameters**			
BMI (kg/m^2^)	24.12 ± 3.24	27.13 ± 3.26	0.000
Total Cholesterol (mmol/L)	5.1 ± 1.01	5.31 ± 1.08	0.000
LDL-c (mmol/L)	1.31 ± 0.33	1.13 ± 0.29	0.000
Uric Acid	306.38 ± 84.71	335.49 ± 89.42	0.000
**Metabolic Parameters**			
Waist Circumference (cm)	82.97 ± 9.67	92.98 ± 8.07	0.000
SBP (mmHg)	130.93 ± 16.83	144.83 ± 19.15	0.000
DBP (mmHg)	79.64 ± 8	85.62 ± 9.68	0.000
FPG (mmol/L)	4.68 ± 1.32	5.8 ± 2.24	0.000
Triglycerides (mmol/L)	1.59 ± 1.6	2.31 ± 2.17	0.000
HDL-c (mmol/L)	2.98 ± 0.84	3.18 ± 0.9	0.000

T-tests were used for continuous variables; Chi-square tests were used for categorical variables. Values are present as means ± SDs or number (no.) and %. Abbreviations: BMI, Body mass index; SBP, systolic blood pressure; DBP, diastolic blood pressure; FPG, fasting plasma glucose; HDL-c, high-density lipoprotein cholesterol; LDL-c, low-density lipoprotein cholesterol.

**Table 2:** Dietary nutrient and flavonoid intakes of the subjects from Harbin Cohort Study on Diet, Nutrition, and Chronic Non-Communicable Diseases (HDNNCDS).

Variable	Control (*n* = 6473)	MetS (*n* = 2635)	*p*-Value
**Nutrient Intake**			
Energy (kcal/day)	2297.17 ± 731.26	2328.96 ± 754.7	0.049
Carbohydrate			
Intake (g/day)	355.97 ± 135.28	360.85 ± 137.48	0.120
Intake (g/kcal)	152.95 ± 21.57	153.2 ± 21.66	0.607
Fat			
Intake (g/day)	72.52 ± 23.89	73.34 ± 25.2	0.145
Intake (g/kcal)	32.67 ± 8.22	32.51 ± 8.11	0.394
Protein			
Intake (g/day)	70.2 ± 29.53	71.33 ± 31.11	0.101
Intake (g/kcal)	30.22 ± 6.22	30.28 ± 6.6	0.673
Fiber			
Intake (g/day)	13.66 ± 6.58	13.76 ± 6.61	0.536
Intake (g/kcal)	6.05 ± 2.41	6.05 ± 2.5	0.975
Copper			
Intake (ug/day)	2433.62 ± 904.96	2454 ± 927.69	0.328
Intake (ug/kcal)	1053.54 ± 181.91	1044.85 ± 193.73	0.048
**Flavonoid intake**			
Total flavonoid			
Intake (mg/day)	34.68 ± 20.88	34.12 ± 20.81	0.241
Intake (mg/kcal)	15.43 ± 8.19	15.04 ± 8.31	0.042
Kaempferol			
Intake (mg/day)	11.34 ± 6.81	11.4 ± 6.9	0.687
Intake (mg/kcal)	5.05 ± 2.7	5.05 ± 2.82	0.981
Luteolin			
Intake (mg/day)	9.08 ± 5.66	8.85 ± 5.54	0.081
Intake (mg/kcal)	4.03 ± 2.18	3.89 ± 2.12	0.004
Isorhamnetin			
Intake (mg/day)	8.32 ± 5.11	8.25 ± 5.07	0.534
intake (mg/kcal)	3.71 ± 2.02	3.65 ± 2.04	0.221
Quercetin			
Intake (mg/day)	3.58 ± 2.21	3.52 ± 2.17	0.236
Intake (mg/kcal)	1.59 ± 0.86	1.55 ± 0.85	0.044
Apigenin			
Intake (mg/day)	2.36 ± 1.54	2.42 ± 1.6	0.086
intake (mg/kcal)	1.05 ± 0.64	1.08 ± 0.69	0.099

Variables are presented in two forms: g/day, which is the crude grams of dietary nutrient and flavonoid intake per day, and g/kcal, which is the grams of dietary nutrient and flavonoid intake standardized by energy. T-tests were used for continuous variables; values are presented as means ± SDs.

**Table 3:** Odd radios (ORs) and 95% confidence intervals (CIs) of MetS risk scores by quartile of energy-adjusted flavonoid and copper intake.

		Q1	Q2	Q3	Q4	P_trend_
Flavonoid	Case/control	712/1594	662/1607	645/1622	616/1650	
	Mode1	1.00	0.87 (0.76–0.99)	0.79 (0.69–0.90)	0.72 (0.63–0.82)	0.000
	Mode2	1.00	0.98 (0.84–1.13)	0.83 (0.72–0.97)	0.77 (0.66–0.90)	0.002
Copper	Case/control	678/1568	681/1602	633/1657	628/1661	
	Mode1	1.00	0.94 (0.83–1.07)	0.83 (0.73–0.95)	0.79 (0.69–0.90)	0.001
	Mode2	1.00	0.95 (0.82–1.11)	0.85 (0.74–0.99)	0.81 (0.70–0.94)	0.020

**Table 4:** ORs and 95% CIs of MetS risk scores by tertile of energy-adjusted flavonoid intake and secondary to energy-adjusted copper intake.

		Q1	Q2	Q3	P_interaction_
Flavonoid	Case/control	932/2105	858/2174	845/2194	0.000
	Model 1	1.00	0.81 (0.73–0.91)	0.75 (0.67–0.85)	
	Model 2	1.00	0.85 (0.75–0.97)	0.80 (0.70–0.91)	
Copper	Case/control	1374/3180	1261/3293		
	Model 1	1.00	0.83 (0.76–0.91)		
	Model 2	1.00	0.85 (0.77–0.95)		

Flavonoid and Cu intakes in the model were first standardized by energy (the crude dietary flavonoids or nutrients intake per 1000 kcal total energy) and then divided into tertile categories and secondary categories based on the cumulative average, respectively. Data are presented as OR and 95% CI of quartile of flavonoid and copper intake. Model 1 adjusted for sex and age, and Model 2 includes Model 1 plus adjusts for BMI, drinking, smoking, and physical activity.

## References

[1] Bonora E., Kiechl S., Willeit J., Oberhollenzer F., Egger G., Bonadonna R.C., Muggeo M., Bruneck Study (2003). Metabolic syndrome: Epidemiology and more extensive phenotypic description. Cross-sectional data from the Bruneck Study. Int. J. Obes. Relat. Metab. Disord..

[2] Sahyoun N.R., Jacques P.F., Zhang X.L., Juan W., McKeown N.M. (2006). Whole-grain intake is inversely associated with the metabolic syndrome and mortality in older adults. Am. J. Clin. Nutr..

[3] Kim J.A., Kim S.M., Lee J.S., Oh H.J., Han J.H., Song Y., Joung H., Park H.S. (2007). Dietary patterns and the metabolic syndrome in Korean adolescents: 2001 Korean National Health and Nutrition Survey. Diabetes Care.

[4] Esmaillzadeh A., Kimiagar M., Mehrabi Y., Azadbakht L., Hu F.B., Willett W.C. (2007). Dietary patterns, insulin resistance, and prevalence of the metabolic syndrome. Am. J. Clin. Nutr..

[5] Lutsey P.L., Steffen L.M., Stevens J. (2008). Dietary intake and the development of the metabolic syndrome: The atherosclerosis risk in communities study. Circulation.

[6] Kandaswami C., Middleton E. (1994). Free radical scavenging and antioxidant activity of plant flavonoids. Adv. Exp. Med. Biol..

[7] Chen G., Li X., Saleri F., Guo M. (2016). Analysis of flavonoids in rhamnus davurica and its antiproliferative activities. Molecules.

[8] Zhang B., Cheng X.R., da Silva I.S., Hung V.W., Veloso A.J., Angnes L., Kerman K. (2013). Electroanalysis of the interaction between (–)-epigallocatechin-3-gallate (EGCG) and amyloid-β in the presence of copper. Metallomics.

[9] Scheiber I., Dringen R., Mercer J.F. (2013). Copper: Effects of deficiency and overload. Met. Ions Life Sci..

[10] Obeid O., Elfakhani M., Hlais S., Iskandar M., Batal M., Mouneimne Y., Adra N., Hwalla N. (2008). Plasma copper, zinc, and selenium levels and correlates with metabolic syndrome components of lebanese adults. Biol. Trace Elem. Res..

[11] Choi M.K., Bae Y.J. (2013). Relationship between dietary magnesium, manganese, and copper and metabolic syndrome risk in Korean adults: The Korea National Health and Nutrition Examination Survey (2007–2008). Biol. Trace Elem. Res..

[12] Temerk Y.M., Ibrahim M.S., Kotb M., Schuhmann W. (2013). Interaction of antitumor flavonoids with dsDNA in the absence and presence of Cu (II). Anal. Bioanal. Chem..

[13] Jabeen E., Janjua N.K., Hameed S. (2014). β-Cyclodextrin assisted solubilization of Cu and Cr complexes of flavonoids in aqueous medium: A DNA-interaction study. Spectrochim. Acta A Mol. Biomol. Spectrosc..

[14] Tan J., Wang B., Zhu L. (2009). DNA binding and oxidative DNA damage induced by a quercetin copper (II) complex: Potential mechanism of its antitumor properties. J. Biol. Inorg. Chem..

[15] Na L., Wu X., Feng R., Li J., Han T., Lin L., Lan L., Yang C., Li Y., Sun C. (2015). The Harbin cohort study on diet, nutrition and chronic non-communicable diseases: Study design and baseline characteristics. PLoS ONE.

[16] He Y.N., Zhai F.Y., Yang X.G., Ge K.Y. (2009). The Chinese diet balance index revised. Acta Nutr. Sin..

[17] Cao J., Chen W., Zhang Y., Zhang Y.Q., Zhao X.J. (2010). Content of selected flavonoids in 100 edible vegetables and fruits. Food Sci. Technol. Res..

[18] Alberti K.G., Zimmet P., Shaw J., IDF Epidemiology Task Force Consensus Group (2005). The metabolic syndrome-a new worldwide definition. Lancet.

[19] Grosso G., Stepaniak U., Micek A., Stefler D., Bobak M., Pająk A. (2017). Dietary polyphenols are inversely associated with metabolic syndrome in Polish adults of the HAPIEE study. Eur. J. Nutr..

[20] Sohrab G., Hosseinpour-Niazi S., Hejazi J., Yuzbashian E., Mirmiran P., Azizi F. (2013). Dietary polyphenols and metabolic syndrome among Iranian adults. Int. J. Food Sci. Nutr..

[21] Rizza S., Muniyappa R., Iantorno M., Kim J.A., Chen H., Pullikotil P., Senese N., Tesauro M., Lauro D., Cardillo C. (2011). Citrus polyphenol hesperidin stimulates production of nitric oxide in endothelial cells while improving endothelial function and reducing inflammatory markers in patients with metabolic syndrome. J. Clin. Endocrinol. Metab..

[22] Schewe T., Steffen Y., Sies H. (2008). How do dietary flavanols improve vascular function? A position paper. Arch. Biochem. Biophys..

[23] Lefevre M., Keen C.L., Lönnerdal B., Hurley L.S., Schneeman B.O. (1986). Copper deficiency-induced hypercholesterolemia: Effects on HDL subfractions and hepatic lipoprotein receptor activity in the rat. J. Nutr..

[24] Salonen J.T., Salonen R., Korpela H., Suntionen S., Tuomilehto J. (1991). Serum copper and the risk of acute myocardial infarction: A prospective population study in men in Estern Finland. Am. J. Epidemiol..

[25] Sitasawad S., Deshpande M., Katdare M., Tirth S., Parab P. (2001). Beneficial effect of supplementation with copper sulfate on STZ-diabetic mice (IDDM). Diabetes Res. Clin. Pract..

[26] Suarez-Ortegón M.F., Ordoñez-Betancourth J.E., Aguilar-de Plata C. (2013). Dietary zinc intake is inversely associated to metabolic syndrome in male but not in female urban adolescents. Am. J. Hum. Biol..

[27] Mira L., Fernandez M.T., Santos M., Rocha R., Florêncio M.H., Jennings K.R. (2002). Interactions of flavonoids with iron and copper ions: A mechanism for their antioxidant activity. Free Radic. Res..

[28] Chun O.K., Chung S.J., Song W.O. (2007). Estimated dietary flavonoid intake and major food sources of U.S. adults. J. Nutr..

[29] Zamora-Ros R., Andres-Lacueva C., Lamuela-Raventós R.M., Berenguer T., Jakszyn P., Barricarte A., Ardanaz E., Amiano P., Dorronsoro M., Larrañaga N. (2010). Estimation of dietary sources and flavonoid intake in a Spanish adult population (EPIC-Spain). J. Am. Diet. Assoc..

